# Linking Tumor Microenvironment to Plasticity of Cancer Stem Cells: Mechanisms and Application in Cancer Therapy

**DOI:** 10.3389/fonc.2021.678333

**Published:** 2021-06-28

**Authors:** Xiaobo Zheng, Chune Yu, Mingqing Xu

**Affiliations:** ^1^ Department of Liver Surgery, West China Hospital, Sichuan University, Chengdu, China; ^2^ Laboratory of Tumor Targeted and Immune Therapy, Clinical Research Center for Breast, State Key Laboratory of Biotherapy, West China Hospital, Sichuan University, Chengdu, China; ^3^ Department of Hepatopancreatobiliary Surgery, Meishan City People’s Hospital, Meishan Hospital of West China Hospital, Sichuan University, Meishan, China

**Keywords:** cancer stem cell, plasticity, tumor microenvironment, cancer progression, resistance

## Abstract

Cancer stem cells (CSCs) are a minority subset of cancer cells that can drive tumor initiation, promote tumor progression, and induce drug resistance. CSCs are difficult to eliminate by conventional therapies and eventually mediate tumor relapse and metastasis. Moreover, recent studies have shown that CSCs display plasticity that renders them to alter their phenotype and function. Consequently, the varied phenotypes result in varied tumorigenesis, dissemination, and drug-resistance potential, thereby adding to the complexity of tumor heterogeneity and further challenging clinical management of cancers. In recent years, tumor microenvironment (TME) has become a hotspot in cancer research owing to its successful application in clinical tumor immunotherapy. Notably, emerging evidence shows that the TME is involved in regulating CSC plasticity. TME can activate stemness pathways and promote immune escape through cytokines and exosomes secreted by immune cells or stromal cells, thereby inducing non-CSCs to acquire CSC properties and increasing CSC plasticity. However, the relationship between TME and plasticity of CSCs remains poorly understood. In this review, we discuss the emerging investigations on TME and CSC plasticity to illustrate the underlying mechanisms and potential implications in suppressing cancer progression and drug resistance. We consider that this review can help develop novel therapeutic strategies by taking into account the interlink between TME and CSC plasticity.

## Introduction

Cancer stem cells (CSCs) are a unique subpopulation of cancer cells that possess self-renewal and differentiation abilities. CSC differentiation enhances the aggressiveness of tumors, thereby aggravating cancer progression (1). CSCs are essential for intratumoral heterogeneity and are responsible for tumor relapse, metastasis, and therapeutic resistance. Moreover, these cells are difficult to eliminate by conventional therapies, rendering additional challenges in cancer management (2). Recently, emerging evidence shows that CSCs can present different phenotypes that render diverse functions with varying degrees of mediating tumorigenesis and progression (3), which is attributed to plasticity of CSCs. Notably, CSC plasticity hinders successful cancer therapies, and it is indeed a pivotal area of research to better understand CSC dynamics and thereby the subsequent development of efficient targeting therapies (4). Although CSCs display a high level of plasticity, how they dynamically transit between non-CSC and CSC states or among varied phenotypes of CSC subsets, and what are the molecular mechanisms underlying these dynamic processes remain poorly understood (5–7). Recently, tumor microenvironment (TME) has been identified as a promising target for cancer therapy, owing to its successful application in clinical tumor immunotherapy. Interestingly, an emerging role of the TME in remodeling CSC plasticity has been observed; the CSC niche is critical in regulating CSC plasticity (8). Within this niche, various cell types, including immune cells, mesenchymal stem cells (MSCs), cancer-associated fibroblasts (CAFs), and exosomes derived from live cells, in addition to the physical and chemical composition of the microenvironment, play roles in maintaining and promoting phenotypic transition of CSCs by secreting factors or providing an immunosuppressive environment (9). In this review, we comprehensively discuss the recent advances with respect to the interaction between TME and CSC plasticity and illustrate the underlying molecular mechanisms. Further, this overview can help provide new insights into the existing therapeutic approaches and designing potential strategies for cancer therapy.

## Emergence of Plasticity of CSCs

Cellular plasticity is the ability of cells to differentiate into multiple lineages, which occurs not only during embryonic development but throughout life (10–12). Although plasticity is a highly regulated process under physiological conditions, cancer cells can utilize this adaptive ability for their survival and progression (13). Recently, several studies have demonstrated that CSCs exhibit varied states and can transition between states dynamically during cancer progression, corroborating CSC plasticity (14–16). Chaffer et al. observed that non-CSCs could spontaneously transit to CSC-like phenotype *in vitro* and *in vivo* in breast cancer cells; this transition was regulated by ZEB1, a key regulator of the epithelial–mesenchymal transition (EMT) (17). Further, Dirkse et al. reported that the well-accepted CSC markers, such as CD133, A2B5, SSEA, and CD15, are not uniformly expressed among glioblastoma cells. Most of the cancer cells adapt a plastic state in response to stimuli in the TME (18). Conclusively, they proposed that CSC plasticity is an adaptation of the cancer cells to the extracellular pressure in the TME, which includes chemical signals, hypoxia-induced physical pressure, or inflammatory environment. In melanoma, JARID1B, a marker of melanoma stem-like cells, was dynamically regulated, indicating the dynamic nature of CSCs (19). Colorectal cancer (CRC), a classical disease model to study CSCs, showed compelling evidence of CSC plasticity during tumor evolution. LGR5, a characteristic marker of CRC stem cells, was expressed in human colon cancer cell lines developed by Kobayashi et al., confirming CSC properties in the established cell lines (20). On treating one of these cell lines with an anticancer drug, they observed transition from LGR5^+^ to LGR5^-^ state, while withdrawal of the drug resulted in the cells reverting to the LGR5^+^ state, indicating the inherent plasticity of CSCs. Interestingly, there are two contradictory opinions on the effect of CSC plasticity on inducing liver metastases: one is that CSC plasticity is primarily associated with tumorigenesis and not cancer metastasis (21), whereas the other considers that a majority of CRC metastases are seeded by CSCs (22). The controversy indicates that the non-CSC-to-CSC transition and plasticity of CSCs are crucial for both primary tumor and metastatic growth. Reportedly, vasculogenic mimicry (VM), a hallmark process of cancer cell switch by which cancer cells transdifferentiate and acquire endothelial cell-like properties, accompanies CSC plasticity (23). Zhang et al. revealed that in renal cell carcinoma, high expression of the CSC markers CD133 and CD44 and VM correlated with poor survival (24). Taken together, CSC plasticity mediates interconversion of CSC subsets, as well as gives rise to non-CSC (differentiated) cells.

## Plasticity of CSCs Contributes to Tumor Heterogeneity

Tumor heterogeneity in cancer biology is widely investigated for efficient clinical management of cancers (25–27). Intrinsic intratumoral heterogeneity and acquired diversification under therapy endow some tumor cells to gain aggressiveness, rendering their survival and emergence of resistance to therapy. These properties are the driving forces for the development of therapy-resistant populations that ultimately result in relapse and metastasis (28, 29). The origin of intratumoral heterogeneity in tumor cells has been widely considered to be resulting from two controversial explanations: clonal evolution and the CSC model (30, 31). In 1976, Peter Nowell first proposed the theory of clonal evolution for tumor heterogeneity, suggesting that step-wise clonal selection is essential for introducing mutations in tumor genes. In this process, a new tumor is derived from a single cell, and poorer tumor outcomes result from multistep mutations, allowing the selection of more aggressive subclones in the derived clonal population. He hypothesized that in the main effective subclone, cells may acquire identical tumorigenic abilities (32). On the contrary, the CSC hypothesis proposes that only a small portion of the tumor subclone have tumorigenic potential and self-renewal ability (31, 33–36). CSCs can differentiate into non-CSCs that develop into a bulk of tumor mass, in a fashion similar to that of stem cell development (37). In 2001, Reya et al. proposed that there exists a minority of subclones with stem cell traits in the tumor tissue, with self-renewal and pluripotent differentiation potential (38). Currently, CSCs have been isolated from more than 10 tumor types, including breast and lung cancers, CRC, melanoma, and glioma (39–42). The origin of tumor cells remains vaguely understood, and tumor heterogeneity augments the difficulties encountered in tumor therapy. Tumor heterogeneity exists objectively, as supported by the fact that some cells are tumorigenic, while others are not (43–46). Therefore, questions over the origin of tumorigenic cells and whether they are CSCs or normal cells still prevail. One opinion is that tumorigenic cells emerge either from stem/progenitor cells or normal somatic cells that acquire mitotic ability (26). Clonal evolution and the CSC model are not necessarily mutually exclusive. Notably, CSC plasticity enhances complexity of intratumoral heterogeneity. However, the question whether CSCs are tumor-initiating cells or primary tumor cells remains to be clarified. The fact that CSCs can change phenotypes *via* different programs, such as dynamic epithelial/mesenchymal status, has led to the speculation that non-CSCs can transform back to CSCs (47–49). Therefore, CSC plasticity can be presumed as the cell state capable of being shaped by EMT, wherein this process can allow interconversion of CSCs and non-CSCs (50–53).

## Influence of TME on CSC Plasticity

For many years, the emphasis of tumor therapy has been on the tumor cells themselves, with a focus on inhibiting their innate ability to adhere and migrate. However, in recent years many studies have shown that tumor cells and peritumor cells (the tumor niche) closely communicate through signaling pathways (54–56). Cells in the tumor niche (such as fibroblasts and immune cells) or cytokines secreted by these cells are accomplices in tumor metastasis and chemoresistance (57). Tumorigenesis and metastasis are closely related to the TME (54), where the niche is not only involved in tissue function, structure, and metabolism but also related to the intracellular milieu of tumor cells (58). The TME can alter the conductions such that they are conducive for tumor growth, survival, and development through autocrine or paracrine secretion (58). Local tissues or distant sites can in turn limit and influence tumorigenesis as well as tumor growth and development through metabolism, secretion, immunity, and structural and functional changes. Both the tumor and the surrounding environment are interdependent and mutually promoting as well as not antagonizing each other. Further, characteristics of the TME, such as immunocyte and mesenchymal cell populations, exosomes, hypoxia, low pH, nutritional deficiencies, and angiogenesis, are key to tumor formation and progression (59, 60).

Emerging evidence suggests that the immunocytes, a critical component in the TME, can regulate phenotypic plasticity of CSCs (61–63). Macrophages, an important cell type involved in complex regulating networks in the TME, are crucial in regulating CSC plasticity. Rao et al. emphasized the mutual influence and interactions between macrophages and CSCs. They reported that CD44 overexpressed by CSCs could induce the macrophages in the TME to secrete the cytokine osteopontin that can in turn bind to CD44 on the surface of tumor cells, thereby promoting tumor cell subclone formation (64). Moreover, analysis of clinical samples showed that osteopontin and CD44 correlated with the survival rate of patients with colon cancer. Additionally, macrophages can secrete oncostatin-M, a pleiotropic cytokine belonging to the IL-6 family, during chemotherapy. Oncostatin-M in turn can activate the dedifferentiation of non-CSCs into aggressive CSCs in triple negative breast cancer (TNBC) (65). Similarly, interplay between macrophage polarization and CSC plasticity can alter the status of cancer cells in terms of EMT, thereby modulating plasticity of stemness in the TME (66). Reportedly, the stem cell factor LIN28, identified in ovarian CSCs, correlates with tumor growth and prognosis of ovarian cancer (67). Using advanced gene sequencing technology, LIN28 and the signaling molecule bone morphogenic protein-4, secreted by macrophages, were observed to be mutually regulated.

CAFs are a predominant component in the TME and play an important role in regulating CSC plasticity (68, 69). CAFs can modulate CSC plasticity through the IGF-II/IGF1R signaling pathway in lung cancer (70); FAK signaling in pancreatic adenocarcinoma (71); and c-Met/FRA1/HEY1 signaling in hepatocellular carcinoma (72). Normal non-cancerous fibroblasts embedded in the TME, upon exposure to chemotherapeutic drugs, undergo DNA damage and secrete a series of cytokines that stimulate cancer growth. Reportedly, the proteoglycan decorin, secreted by fibroblasts, inhibits tumor growth and can induce the expression of tumor-suppressor genes in the microenvironment surrounding TNBC, thereby restraining tumor metastasis (73). GATA3 can also inhibit cancer metastasis and is aberrantly expressed or deleted in most patients with breast cancer (74). Moreover, GATA3 can activate the downstream molecule miR-29b that can further inhibit the synthesis of proteins required for tumor metastasis. In the absence of GATA3, the metastasis of cancer cells cannot be stopped, and metastatic tumor cells can induce inflammatory responses, stimulate angiogenesis, and acquire nutrients for metastasis. Nakasone et al. observed increased sensitivity of breast cancer cells to drugs in mice after the selective deletion of two distinct types of TME factors, MMP9 and CCR2 (75). Moreover, treatment with HGF or combinatorial inhibition of RAF and MET can be used as potential therapeutic strategies in *BRAF*-mutant melanoma. MSCs, which are mature progenitor cells, are essential components of the TME and considered to assist in metastasis (76). Following contact with MSCs, breast cancer cells activate lysyl oxidase expression that can enhance their metastatic ability and promote primary tumor dissemination to the lungs and bones. In a recent study, NOTCH1 signaling activated by MSC-derived dermal fibroblasts was observed to regulate plasticity and stemness of melanoma stem/initiating cells (77). This finding suggests that CAF-targeted strategies may aid in efficiently eradicating CSCs.

Recent studies have shown that exosomes derived from tumor cells or non-tumor cells are prominent messengers in regulating CSC plasticity (78, 79). For instance, exosomes secreted from CAF contribute to CSC proliferation and induce chemoresistance in colorectal cancer (80). Exosomes also play an important role in tumor metastasis through the premetastatic niche formation (81, 82). Exosomes secreted by stromal cells within the TME facilitate the transformation of non-CSCs into CSCs (83). Hu et al. demonstrated that CAF-derived exosomes significantly promote clonogenicity and increase the percentage of colorectal CSCs by activating the WNT pathway (80). Furthermore, exosomes can regenerate stem cell phenotypes by regulating the stem cell-related signaling pathways, including the Notch pathway, Wnt pathway, and Hedgehog pathway (84). In addition, exosomes derived from CSCs promote the proliferation and metastasis of clear cell renal cell carcinoma by transporting miR-19b-3p (85). Colorectal CSC-derived exosomes also facilitate tumorigenesis through mediating neutrophils (86).

The physical and chemical composition of the CSC niche, such as hypoxia and acidity, can also contribute to the regulation of CSC plasticity (87, 88). It is known that hypoxia modulates various aspects of cancer development and progression, including CSC plasticity. Reportedly, hypoxia could increase the plasticity of CSCs in glioblastoma by upregulating important molecules related to stem cell pathways, such as OCT4, NANOG, and c-MYC (89). The hypoxic niche can also determine the fate of CSCs *in vivo*. Tumor cells in the hypoxic niche show enhanced CSC properties compared to those in the non-hypoxic niche, which is attributed to activation of the ROS/HIF−1α/c−Met pathway (90). Similarly, tumor-derived acidosis can also promote the invasion and metastasis of tumor cells *via* metabolic reprogramming (91). Furthermore, the acidic TME can facilitate immune invasion by inhibiting the activation of effector T cells and inducing M2 macrophage polarization (91, 92). Estrella et al. found that survival of CSCs depends on low pH environments that promote autophagy (93). Furthermore, Spugnini et al. demonstrated that a highly acidic TME can lead to chemoresistance, and targeted proton pumps with inhibitors can improve anti-tumor responses (94). Additionally, accumulating evidence demonstrated that the release of exosomes is significantly improved in an acidic TME, thereby leading to malignant tumor phenotypes (95–97). Collectively, CSCs are the key players in tumor recurrence and metastasis, wherein the TME provides conditions favorable for the growth of CSCs.

## Therapeutic Strategies Encompassing Plasticity and Niche of CSCs

Intratumoral heterogeneity and complexity of the TME are the major challenges in effective cancer treatment. CSC plasticity augments tumor heterogeneity, further enhancing and rendering difficulty in regulating drug resistance, relapse, and metastasis (98). Although chemotherapeutics can eliminate most tumor cells, a minority of CSCs and resistant cancer cells tend to escape the lethal effect of these drugs, eventually rendering tumor recurrence (1). It is even more difficult to completely eradicate CSCs with plasticity. Anticancer drugs normally only target tumor cells within their cell cycle; as plasticity of CSCs varies between the stationary and dynamic states, they are not affected, which is the primary reason for treatment failure (99). Consequently, CSC plasticity is now recognized as a major “target cell population” in oncology (4). Recent studies have shown that therapies targeting both plasticity and niche of CSCs may be promising strategies in suppressing tumor progression. For example, CAFs could activate stem cell pathways and are highly abundant in the TME. Targeting the relevant CAF–CSC signaling axis should therefore eliminate CSCs *via* induced differentiation and/or promoted apoptosis, contributing to tumor regression. Recently, a neutralizing monoclonal antibody against GPR77 was observed to effectively control tumor formation and reverse chemoresistance by eliminating CD10^+^ GPR77^+^ subpopulation of CAFs, proposing a CAF-targeted therapeutic strategy (100). Another research reported that CCL2 mediates a crosstalk between cancer cells and stromal fibroblasts that regulates breast CSCs. CCL2 secreted by CAFs activates the NOTCH1 pathway that further induces CSC phenotype in the breast cancer cells, granting them self-renewal potential, indicating CCL2 as a potential target to block non-CSC-to-CSC switch (101). Luo et al. observed that co-inhibition of glycolysis and thioredoxin and glutathione antioxidant pathways suppresses tumor-initiating potential, tumor growth, and metastasis of breast cancer cells under metabolic stress or hypoxia. The probable reason is that the combination strategy eliminates both quiescent mesenchymal-like and proliferative epithelial-like states of breast CSCs (102). Further, programmed cell death protein 1 blockade, combined with a granulocyte-macrophage colony-stimulating factor-modified CSC vaccine, was observed to enhance a specific antitumor immunotherapy response against bladder cancer (103). Exosomes, hypoxia, and acidity are indeed pivotal for CSC-niche development, and molecules capable of targeting exosomes or acidity or inducing hypoxia are potential therapeutic regimens for eliminating CSCs by reprograming the TME.

CSCs can gain or lose stemness and switch their status by adapting to the physical conditions (hypoxia and acidity), and communicate with stroma and immune cells, corroborating the crosstalk between intrinsic CSC plasticity and niche complexity. Therefore, combination strategies that target CSC plasticity together with immunotherapy or TME-modulating agents could be promising in inhibiting tumor progression and metastasis. However, whether CSC plasticity arises as a consequence of the microenvironment-exerted selection pressure or whether it is an intrinsic, default feature of cancer cells that enables them to adapt to varying conditions of the TME remains poorly unknown. Although most cancer cell subpopulations are capable of phenotypic transition, they vary in their speed and ability of adaptation. Recent studies on glioblastoma suggest that the intrinsic plasticity of tumor cells renders them to randomly switch between different phenotypes (defined by varied expression of CSC markers) and adapt to the TME (18, 104). In summary, these studies underscore that alterations in the CSC niche play important roles in restraining plasticity of CSCs and highlight the need to better understand the crosstalk between TME and CSC plasticity. Collectively, targeting CSCs and their niches is a promising strategy for efficient cancer therapy.

## Conclusions

In this review, we present the recent advances in oncology that relate TME and CSC plasticity. All the observations pinpoint to the fact that developing novel CSC plasticity-suppressing strategies by targeting TME can improve cancer prognosis and patient survival. Therefore, we propose that exploiting the intrinsic dependence of CSCs to interact with non-tumor cell types in the CSC niche is a potential strategy for cancer therapy. The need-of-the-hour is, therefore, to understand the fundamental mechanisms underlying CSC plasticity and to illustrate the effect of the dynamic properties of CSCs; these aspects can subsequently help improve clinical management of cancers. Further investigation on the interactions of CSC plasticity, tumor, and TME, particularly clarifying the associated signaling pathways, will greatly facilitate our understanding of the invasive and metastatic features of malignant tumors. Regarding application in clinical treatment, combination TME-targeted therapy with the molecular drugs that reverse or block CSC plasticity should be envisaged to provide new insights into effectively inhibiting tumor metastases and efficiently managing cancer. Finally, this combination therapy can be applied in neoadjuvant chemotherapy and postsurgical resection, to help eradicate residual, dormant, and distantly located CSCs, potentially preventing distant metastases.

## Author Contributions

MX proposed and supervised the research. XZ and CY collected the references. XZ, and CY drafted the paper. All authors contributed to the article and approved the submitted version.

## Funding

This study was supported by grants from the National Natural Science Foundation of China (No. 81803574), China Postdoctoral Science Foundation (2019M653430), Post-Doctor Research Project, West China Hospital, Sichuan University (2018HXBH003), and Key Technology Research and Development Program of the Sichuan Province (2019YFS0208, 2021YFSY0009).

## Conflict of Interest

The authors declare that the research was conducted in the absence of any commercial or financial relationships that could be construed as a potential conflict of interest.

## Abbreviations

CAFs, cancer associated fibroblasts; CRC, colorectal cancer; CSCs, cancer stem cells; EMT, epithelial–mesenchymal transition; MSCs, mesenchymal stem cells; TME, tumor microenvironment; TNBC, triple negative breast cancer; VM, vasculogenic mimicry.
